# Influence of Glutamine and Branched-Chain Amino Acids Supplementation during Refeeding in Activity-Based Anorectic Mice

**DOI:** 10.3390/nu12113510

**Published:** 2020-11-14

**Authors:** Clément L’Huillier, Marine Jarbeau, Floriane Pingeon, Wafa Bahlouli, Emmeline Salameh, Asma Amamou, Alexis Goichon, Pauline Tirelle, Jean-Luc do Rego, Pierre Déchelotte, Moïse Coëffier

**Affiliations:** 1 Normandie University, UNIROUEN, INSERM UMR 1073 Nutrition, Inflammation and Gut-Brain Axis, 76000 Rouen, France; cle.lhuillier@gmail.com (C.L.); jarbeau.marine@laposte.net (M.J.); floriane.pingeon@outlook.fr (F.P.); wafa.bahlouli@etu.univ-rouen.fr (W.B.); emmeline.salameh@gmail.com (E.S.); asma.amamou@etu.univ-rouen.fr (A.A.); alexis.goichon@univ-rouen.fr (A.G.); pauline.tirelle@etu.univ-rouen.fr (P.T.); pierre.dechelotte@chu-rouen.fr (P.D.); 2 Institute of Research and Innovation in Biomedicine (IRIB), UNIROUEN, 76000 Rouen, France; jean-luc.do-rego@univ-rouen.fr; 3 Animal Behavior Facility, SCAC, UNIROUEN, 76000 Rouen, France; 4 Department of Nutrition, Rouen University Hospital, 76000 Rouen, France

**Keywords:** activity-based anorexia, glutamine, branched-chain amino acids, refeeding, protein synthesis, colon

## Abstract

Background: Optimizing the refeeding of patients with anorexia nervosa remains important to limit somatic complications of malnutrition, as well as to avoid disease relapses by targeting persistent mood and intestinal disorders. We aimed to evaluate the effects of glutamine (Gln) and branched-chain amino acids (BCAA) supplementation during refeeding in activity-based anorectic (ABA) mice. Method: Male C57Bl/6 mice were randomized in control and ABA groups. Once ABA-induced malnutrition was established, mice were progressively refed or not. Refed mice had free access to drinking water supplemented or not with 1% Gln or 2.5% BCAA for 10 days. Results: A progressive refeeding was associated with a partial restoration of body weight and lean mass, while a fat mass rebound was observed. In addition, refeeding restored glucose and leptin. Gln did not affect these parameters, while BCAA tended to increase body weight, fat mass, and glycaemia. In the colon, refeeding improved total protein synthesis and restored the LC3II/LC3I ratio, a marker of autophagy. Gln supplementation enhanced colonic protein synthesis, which was associated with an increased p-p70S6kinase/p70S6kinase ratio, whereas these effects were blunted by BCCA supplementation. Conclusions: In ABA mice, Gln and BCAA supplementations during a progressive refeeding fail to restore body weight and lean mass. However, Gln supplementation improves total colonic protein synthesis conversely to BCAA. Further studies are needed to decipher the underlying mechanisms involved in these opposite results.

## 1. Introduction

Anorexia nervosa (AN), an eating disorder (ED) mainly characterized by a severe weight loss (BMI < 18.5 kg/m^2^), an intense fear of weight gain or persistent behavior impairing weight restoration, and alteration of body shape perception [1], predominantly affects young women with more than 75% of diagnoses set before 22 years [2]. Its mean lifetime prevalence is 1.4% in women versus 0.2% in men [3]. AN is a multifactorial disease involving social, genetic, and psychological [4] factors, but its etiology remains relatively poorly-understood. Due to its comorbidities such as anxiety and depression [5,6], mortality reaches 1–4% [7]. Although early medical care is reported to improve the probabilities of complete recovery [8], relapse occurs in 31% of patients after discharge [9]. In addition, the gastrointestinal tract is deeply affected in AN patients who exhibit delayed gastric emptying, intestinal motility disorders, bloating, abdominal pain, and gut dysbiosis [10,11]. Clinical guidelines recommend refeeding AN patients through an enteral route [12] via a nasogastric probe. Despite the safety and efficiency of this refeeding protocol, recovery remains incomplete. Indeed, after 7 months, Haas et al. reported that protein mass is still lower than the standard baseline [13], while body weight recovery is associated with an increased truncal fat mass [14]. Moreover, intestinal complaints and mood disorders are still present after weight restoration [15,16]. For instance, in the activity-based anorexia (ABA) murine model, which shares some common features with AN patients such as physical hyperactivity [17], altered fat and lean mass, and reduced gastric emptying [18] refeeding failed to restore intestinal permeability and was associated with a marked increase of fat mass [19]. These data highlight the need for new refeeding strategies to improve recovery and limit associated comorbidities.

For this purpose, supplementation with some specific amino acids could be of interest. On the one hand, it has been demonstrated that branched-chain amino acids (BCAA) were preferentially metabolized in the skeletal muscle [20], leading to an increase in lean mass during intense physical activity both in mice [21] and humans [22,23] or maintaining it in older subjects [24], suggesting that BCAA could be useful to improve body composition in AN patients during refeeding. On the other hand, the beneficial effects of glutamine (Gln) on the intestinal barrier function [25,26], protein synthesis [27], and intestinal inflammatory response [28] have been reported. Interestingly, in a recent randomized double-blind clinical trial, Zhou et al. observed that Gln improves intestinal disorders and reduces intestinal permeability in patients with irritable bowel syndrome [29]. In a recent study, we showed that Gln, but not BCAA, was able to restore paracellular permeability and protein synthesis in the colon of ABA mice. By contrast, both BCAA and Gln failed to improve body weight and body composition [30]. However, these data have been obtained in restricted mice. Therefore, we hypothesized that insufficient macronutrients and energy intake blunted the putative beneficial effects of BCAA and Gln.

## 2. Materials and Methods

### 2.1. Animal Procedure

A protocol was approved by the regional ethical committee named CENOXEMA (authorization on APAFIS#9473-2016072016008305 v6). Eight week-old male C57Bl/6 mice (Janvier Labs, Le Genest-Saint-Isle, France, *n* = 88) were placed in individual cages and fed with an ad libitum standard diet and water. After an acclimatizing period of 5 days at 23 °C with a reversed 12 h dark-light cycle (dark phase: From 10:00 a.m. to 10:00 p.m.), mice were randomized and separated in two groups: Control (CTRL) and activity-based anorexia (ABA). Only ABA cages were equipped with an activity wheel and connected with the Running Wheel^®^ software (Intellibio, Seichamps, France), while CTRL mice were placed in standard cages. The ABA procedure was performed as previously described [31]. Briefly, after 5 days of acclimatization with the activity wheel, food access was progressively limited from 6 h at day 6 to 3 h at day 9 and until day 13. According to the ethical procedure, animals with a weight loss exceeding 20% in 3 consecutive days were euthanized. Body weight, food intake, and wheel activity were monitored daily before the dark phase.

At day 14, ABA mice were split into four sub-groups: 1/No refeeding (ABA-C); 2/progressive refeeding (ABA-Rf); 3/progressive refeeding with Gln supplementation (ABA-Rf-G), and 4/progressive refeeding with BCAA supplementation (ABA-Rf-B). Progressive refeeding started at day 14 and consisted of an increase in the food amount of 0.5 g/day from 2.5 g at day 14 to 5 g at day 19 and until the end of the protocol at day 23. Gln and BCAA supplementations, assessed in two distinct experiments, were given in drinking water as previously described [30]. Briefly, the dose of Gln was calculated to reach 2 g/kg/day. The BCAA beverage consisted of a combination of Leucine (Leu), Isoleucine (Ile), and Valine (Val) with a ratio of 2:1:1 (Leu, Ile, Val) and a final concentration of 2.5%. Gln and BCAA solutions were renewed each day and the daily fluid intake was measured by weighing drinking bottles.

At day 23, 20 min before euthanasia with the ketamine/xylazine solution, mice were intraperitoneally injected with 100 µL of PBS containing puromycin (Sigma-Aldrich, Saint Quentin Fallavier, France) at the dose of 0.040 µmol/g to evaluate tissue protein synthesis. Blood samples were collected in heparine-coated collecting tubes, which were centrifuged for 20 min at 3000× *g* at 4 °C. Then, the plasma was removed and stored at −80 °C. All collected samples (colon, hypothalamus, muscles) were immediately dropped in liquid nitrogen.

### 2.2. Gastric Emptying

Gastric emptying was measured following the same protocol used in a previous study [18]. In summary, at day 17, eight CTRL and ABA-C mice were fasted during 12 h with free access to water. Then, mice were placed in an individual cage without an activity wheel and each received a weighed standard food amount for 1 h. Food and water were removed for 1.5 h and the remaining food was weighed in order to calculate the food intake. Animals were euthanized as described previously and after clamping the esophagus at the cardia and pylorus, the stomach was removed and its content was dried and weighed. Gastric emptying was measured by using the formula: Gastric emptying (%) = [1 − (weight of food remaining in the stomach/weight of food intake)]/100. At day 23, this protocol was applied to all remaining CTRL, ABA-Rf, and ABA-Rf-G animals.

### 2.3. Body Composition

The whole body composition was evaluated at day 23 on vigil animals using Minispec LF110 (Brucker, Wissembourg, France), as previously described [30].

### 2.4. Intestinal Permeability

Colonic paracellular permeability was assessed by measuring fluxes of FITC-Dextran (4 kDa) in Ussing chambers (Harvard Apparatus, Holliston, MA, USA), as previously described [32]. The solution in the serosal side was collected after 3 h at 37 °C and the FITC-Dextran fluorescence was measured.

### 2.5. Protein Extraction and Western Blotting

Protein extraction and Western blotting were performed as previously described [30]. Briefly, proteins were extracted by using 400 µL of an ice-cold buffer and homogenization with TissueLyser LT (Qiagen, Courtaboeuf, France) was followed by a 20 min incubation on ice and centrifugation for 15 min at 12,000× *g* at 4 °C. Total protein extracts (25 µg) were loaded on 4–20% gradient polyacrylamide gels for 1 h 30 min at 100 V and then transferred on a nitrocellulose membrane. Protein levels were normalized with β-actin or with GAPDH for muscles. The antibodies data are listed in Supplementary Table S1.

### 2.6. Protein Synthesis Analysis by the SUnSET Method

Surface sensing of translation (SUnSET) is a non-radioactive method used to evaluate the in vivo protein synthesis rate based on a puromycin incorporation level, as previously described [30]. Briefly, puromycin incorporation was assessed by Western blotting using a specific mouse monoclonal anti-puromycin antibody (Supplementary Table S1). Then, lanes were analyzed by densitometry and results were expressed as the ratio puromycin expression/min.

### 2.7. RT-qPCR

Extraction, quantification, and reverse transcription were performed on a colonic and hypothalamic sample, as previously described [33]. The qPCR was realized by using the SYBR Green technology on a Bio-Rad CFX96 real-time PCR system (Bio-Rad Laboratories, Marnes la Coquette, France) for the following targets: MUC-2, CLDN-1, CLDN-2, ZO-1, OCLDN, NPY, POMC, IL-10, TNF-α, MCP-1, CRH, IL-1β, GAPDH, β2M, RPS18 (Supplementary Table S2). Values were obtained from the cycle threshold using the mRNA quantification based on standard curves. The mean of housekeeping genes GAPDH, β2M, and RPS18 was calculated and used as a reference.

### 2.8. Plasma Adiponectin and Leptin

Quantification of plasma adiponectin (KMP0041) and leptin (KMC2281) was performed with ELISA kits (Invitrogen, Carlsbad, CA, United States) following the supplier’s instructions.

### 2.9. Plasma Biochemical Dosages

Albumin, glucose, total cholesterol, and triglycerides quantification from plasma samples were completed on a Catalyst One device (IDEXX Laboratories, Westbrook, ME, USA) using specific chips in accordance with the supplier’s instructions.

### 2.10. Statistical Analysis

Data were analyzed with GraphPad Prism 5.0 (GraphPad Software Inc., San Diego, CA, USA) and expressed as the mean ± standard error mean (SEM). Non-parametric Mann-Whitney tests were used to compare the two groups. Comparisons between more than two groups were performed by using one-way ANOVA followed by Tukey’s tests; or two-way ANOVA followed by Bonferroni as a post-hoc test (for body weight follow-up, Supplementary Figure S1). A value of *p* < 0.05 was considered significant.

## 3. Results

### 3.1. Body Composition and Muscular Protein Synthesis

**Figure 1:**
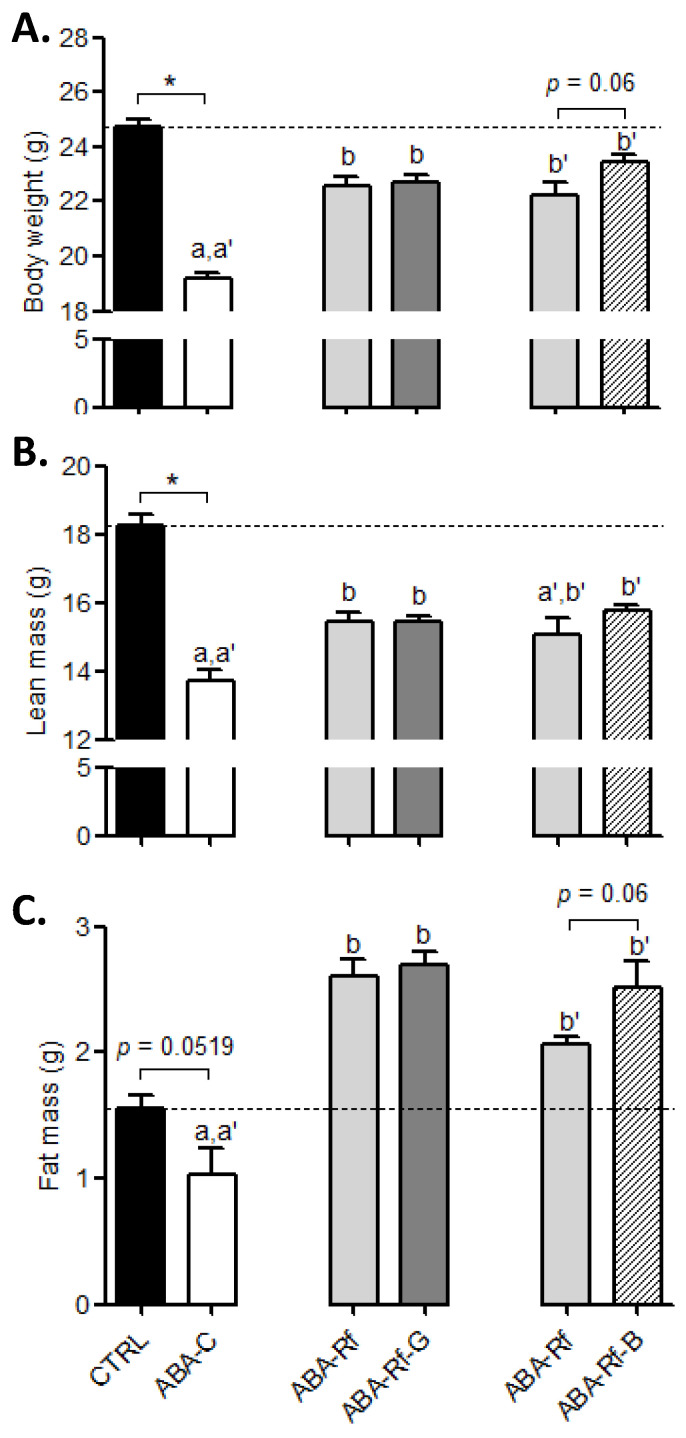
Body weight, lean mass, and fat mass. Body weight (**A**), lean mass (**B**), and fat mass (**C**) measured at day 23 in control mice (CTRL), in activity-based anorexia (ABA) mice (ABA-C), and in ABA mice after refeeding supplemented or not (ABA-Rf group) with 1% glutamine (Gln, ABA-Rf-G group) or 2.5% branched-chain amino acids (BCAA, ABA-Rf-B group). Data from CTRL and ABA-C groups were compared by the Mann-Whitney test, * *p* < 0.05. Then, ABA-C and refed groups were compared with ANOVA followed by Tukey’s post-tests. Values without a common letter (a, b for Gln experiments, and a’, b’ for BCAA experiments) significantly differ (*p* < 0.05). *N* = 5–8 per group.

First, as observed in our previous studies [31,32,34], body weight dropped after food limited access in all ABA groups and then increased when the refeeding phase began (Supplementary Figure S1A,B). At day 23, a pronounced body weight loss was observed in ABA-C mice (−22.5% vs. CTRL), while body weight was partially restored in all refed ABA groups compared to the CTRL mice (Figure 1A). Body weight gain induced by refeeding remained significantly unaffected by both Gln and BCAA. Nevertheless, body weight gain tended to be slightly increased in ABA-Rf-B mice when compared to its own ABA-Rf group (+1.06 g, *p* = 0.06). As observed in anorectic patients, both lean and fat mass were reduced in the ABA-C group when compared to the control (−24.9% and −33.9%, respectively, Figure 1B,C), even if the difference did not reach significance for fat mass (*p* = 0.0519). Although refeeding increased lean mass compared to the ABA-C mice, it was still lower than in the CTRL mice. No difference was observed in lean mass between all ABA refed groups (Figure 1B), suggesting that neither Gln nor BCAA supplementation impacted lean mass during refeeding. In accordance with these results, neither Gln nor BCAA supplementation affected the total muscular protein synthesis (Supplementary Figure S2A). During refeeding, fat mass markedly increased in all refed ABA mice to achieve a higher level than in the CTRL group (Figure 1C). Both Gln and BCAA supplementations showed no significant effect on fat mass gain, even if BCAA seemed to slightly increase fat mass when compared to its own ABA-Rf group (*p* = 0.06). Given that no differences were observed neither between CTRL groups of Gln and BCAA series, nor in their respective ABA-Rf groups, therefore, we chose to pool data for ABA-Rf groups. Finally, in agreement with our first study, we did not observe any effect of amino acids on fluid intake (data not shown).

### 3.2. Gastric Emptying and Hypothalamic Control of Food Intake and Neuropeptides

**Figure 2:**
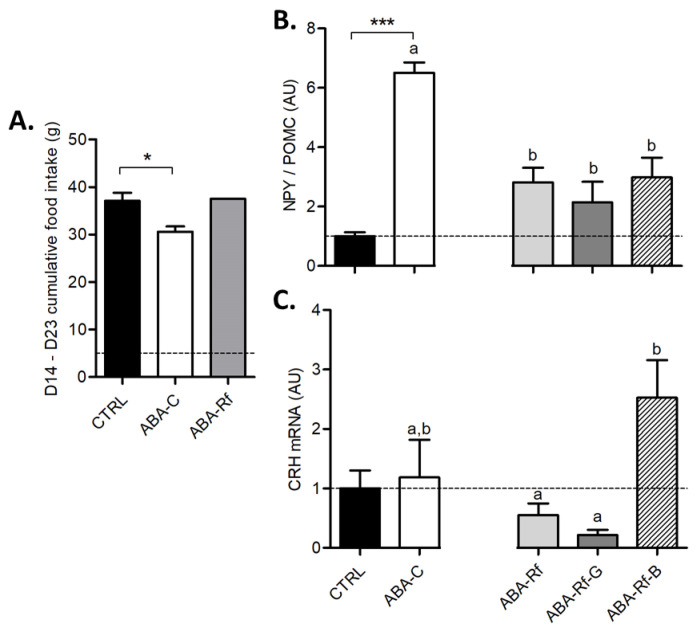
Food intake and hypothalamic mRNA levels. (**A**) Cumulative food intake during the refeeding period (from day 14 to 23) in control mice (CTRL), in activity-based anorexia (ABA) mice (ABA-C), and in ABA mice during refeeding (ABA-Rf groups). The NPY/POMC ratio (**B**) and CRH mRNA level (**C**) measured at day 23 in the hypothalamus in control mice (CTRL), in activity-based anorexia (ABA) mice (ABA-C), and in ABA mice after refeeding supplemented or not (ABA-Rf group) with 1% glutamine (Gln, ABA-Rf-G group) or 2.5% branched-chain amino acids (BCAA, ABA-Rf-B group). Data from CTRL and ABA-C groups were compared by the Mann-Whitney test, * *p* < 0.05 and *** *p* < 0.001. Then, ABA-C and refed groups were compared with ANOVA followed by Tukey’s post-tests. Values without a common letter significantly differ (*p* < 0.05). *N* = 5–8 per group.

Food intake was reduced in the ABA-C group compared with CTRL (Figure 2A). As refeeding consisted of a progressive increase in food availability, food intake was increased in all refed ABA groups compared with ABA-C leading to a similar cumulative food intake (from d14 to 23) in CTRL and refed ABA groups (Figure 2A). Concerning gastric emptying, a slight decrease of 5% was observed in the ABA-C group and was restored in all refed groups without an additional effect of Gln (Supplementary Figure S3). Then, we measured mRNA levels of both NPY and POMC using RT-qPCR to calculate the NPY/POMC ratio, which was found nearly 6 times higher in ABA-C mice than in the CTRL group (Figure 2B). In all refed ABA groups, the NPY/POMC ratio was diminished compared to the ABA-C group, but still remained almost 3 times higher than in the CTRL group without any effects of amino acids supplementation (Figure 2B). Moreover, we evaluated the CRH mRNA level in the hypothalamus. The CRH mRNA level remained unaffected in all groups except in the ABA-Rf-B group (Figure 2C), which exhibited a marked increase.

### 3.3. Plasma Biochemical Dosages

**Figure 3:**
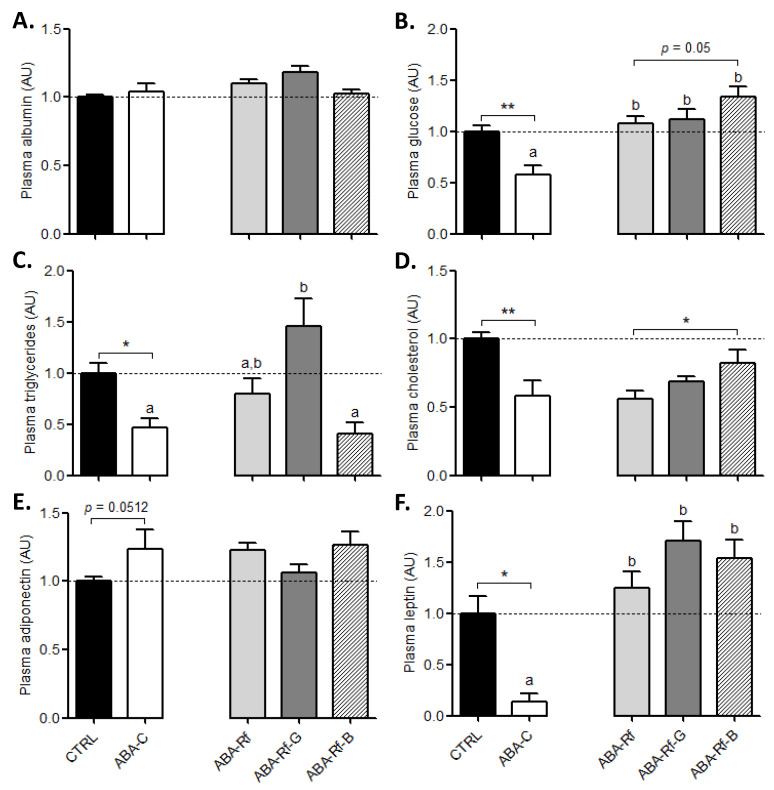
Biological plasma parameters. Albumin (**A**), glucose (**B**), triglycerides (**C**), total cholesterol (**D**), adiponectin (**E**), and leptin (**F**) measured at day 23 in the plasma in control mice (CTRL), in activity-based anorexia (ABA) mice (ABA-C), and in ABA mice after refeeding supplemented or not (ABA-Rf group) with 1% glutamine (Gln, ABA-Rf-G group) or 2.5% branched-chain amino acids (BCAA, ABA-Rf-B group). Data from CTRL and ABA-C groups were compared by the Mann-Whitney test, * *p* < 0.05 and ** *p* < 0.01. Then, ABA-C and refed groups were compared with ANOVA followed by Tukey’s post-tests. Values without a common letter significantly differ (*p* < 0.05). *N* = 5–8 per group.

We analyzed some plasma markers to evaluate whether differential effects of amino acids supplementation were observed at the systemic level. Due to the few amounts of plasma harvested from ABA mice, we decided to focus only on the following markers. We did not observe any significant modification of plasma albumin (Figure 3A), a high molecular weight protein involved in the maintenance of osmotic pressure, which is routinely used as a marker of protein homeostasis. Glycaemia displayed a drop in ABA-C mice compared to the CTRL group, but was restored in all refed ABA groups. Amino acids supplementation did not significantly affect glycaemia restoration induced by refeeding, although BCAA tended to increase it (Figure 3F). Plasma triglycerides concentration was markedly decreased in ABA-C mice, but was partially restored in ABA-Rf mice when compared to the CTRL group (Figure 3C). Gln supplementation was associated with an increase of plasma triglycerides, whereas BCAA blunted the refeeding effect and took back plasma triglycerides to the ABA-C level (Figure 3C). Total plasma cholesterol was also reduced by nearly 50% in the ABA-C group in comparison to the CTRL group. Gln supplementation had no significant effect, whereas BCAA increased plasma total cholesterol compared with ABA-Rf mice (Figure 3D). Finally, we analyzed two adipokines: Adiponectin and leptin. Plasma adiponectin tended to be increased in the ABA-C group compared to the CTRL mice (Figure 3E, *p* = 0.0512). The ABA-Rf group did not show any impact of refeeding on the plasma adiponectin level. Only Gln tended to restore plasma adiponectin but the difference did not reach significance. Leptin was dramatically dropped in ABA-C mice compared to the CTRL group, while it was restored in all refed ABA groups without an effect of Gln or BCAA (Figure 3F).

### 3.4. Colonic Protein Synthesis, Tight Junction Proteins Expression, and Inflammatory Markers

**Figure 4:**
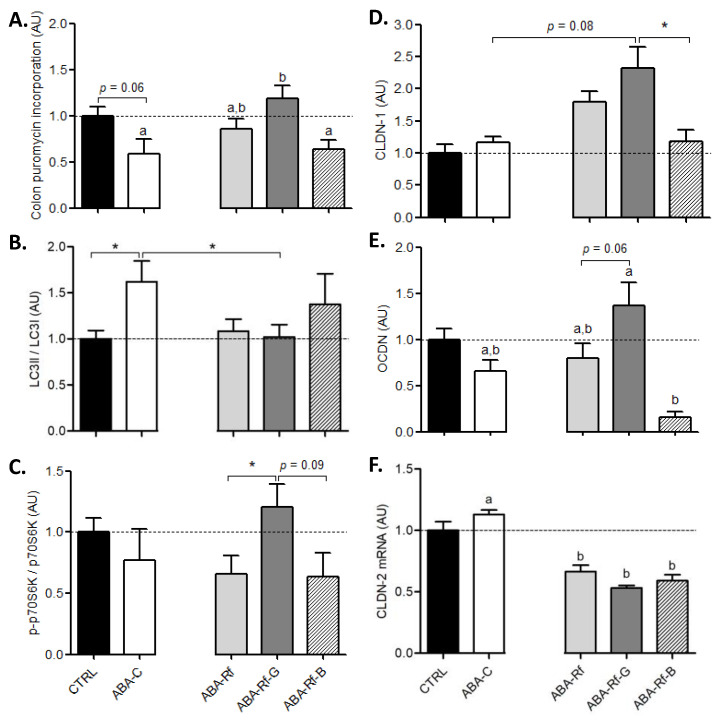
Colonic parameters. Protein synthesis (**A**), LC3II/LC3I ratio (**B**), phosphorylation status of p70S6kinase (**C**), claudin-1 (CLDN-1) (**D**), occludin (OCDN) (**E**), and claudin-2 (CLDN-2) and (**F**) mRNA expression measured at day 23 in the colonic mucosa in control mice (CTRL), in activity-based anorexia (ABA) mice (ABA-C), and in ABA mice after refeeding supplemented or not (ABA-Rf group) with 1% glutamine (Gln, ABA-Rf-G group) or 2.5% branched-chain amino acids (BCAA, ABA-Rf-B group). Data from CTRL and ABA-C groups were compared by the Mann-Whitney test, * *p* < 0.05. Then, ABA-C and refed groups were compared with ANOVA followed by Tukey’s post-tests. Values without a common letter significantly differ (*p* < 0.05). *N* = 5–8 per group.

Then, we focused on colonic response, as previously reported, in which paracellular permeability, inflammatory response, and protein metabolism were altered in the colon of ABA mice [31,32,35]. Total colonic protein synthesis, measured by evaluating the level of in vivo puromycin incorporation (Figure 4A), tended to be decreased in ABA-C mice when compared to the CTRL group (*p* = 0.0573). Refeeding partially restored the colonic protein synthesis, but the difference did not reach significance between ABA-C and ABA-Rf groups. By contrast, Gln supplementation was associated with a significant increase of protein synthesis compared to the ABA-C group (*p* < 0.05). Surprisingly, the ABA-Rf-B group exhibited a low protein synthesis rate at a similar level to the ABA-C group, suggesting that BCAA blunted the effects of refeeding.

As previously reported, the reduced protein synthesis was associated with an increase in autophagy in the colon mucosa of ABA female mice. Moreover, we evaluated the LC3II/LC3I ratio, a marker of autophagy activation (Figure 4B). Interestingly, the LC3II/LC3I ratio was increased in ABA-C mice compared to CTRL (1.6-fold change) and was normalized in both ABA-Rf and ABA-Rf-G groups. The ABA-Rf-B group exhibited an intermediate level of LC3II/LC3I ratio, but the difference did not reach significance. It is well established that both protein synthesis and autophagy are partly regulated by the mTOR pathway. Therefore, we analyzed the phosphorylation status of p70S6 kinase as a marker of the mTOR pathway activation. The ratio p-p70S6K/p70S6K tended to be reduced in ABA-C group compared to the CTRL mice, but the difference was not significant (−22%). Unexpectedly, the p-p70S6K/p70S6K ratio remained diminished in both ABA-Rf and ABA-Rf-B groups. Conversely, only the ABA-Rf-G mice showed an increase of the p-p70S6K/p70S6K ratio beyond the CTRL group. Therefore, the p-p70S6K/p70S6K ratio was significantly higher in ABA-Rf-G than in the ABA-Rf group.

In previous studies [25,31], ABA male mice exhibited at day 17 an increase in colonic paracellular permeability. In the present study, we assessed colonic permeability by measuring FITC-Dextran 4kDa flux through a fresh colon sample in Ussing chambers at day 23. Surprisingly, no difference was found between all groups (data not shown). Despite this, some differences appeared in colonic tight junction proteins expression. CLDN-1, CLDN-2, and OCDN expression were not significantly affected in ABA-C compared with CTRL (Figure 4D–F), although a trend was observed for the OCDN increase. Refeeding was associated with an increase in CLDN-1 expression and a decrease in CLDN-2 mRNA expression, while OCDN remained unaffected. Interestingly, while both Gln and BCAA supplementations did not modify CLDN-2 mRNA expression, the ABA-Rf-G group exhibited a trend for increased CLDN-1 and OCDN expression (Figure 4D,E). By contrast, the ABA-Rf-B group showed a low expression of CLDN-1 and OCDN compared to ABA-Rf and ABA-Rf-G groups. Therefore, Gln supplementation was associated with a higher expression of CLDN-1 and OCDN than BCAA supplementation. Moreover, we assessed mRNA levels for CLDN-1, OCDN, and ZO-1. However, we did not observe major differences (Supplementary Figure S2).

Then, we aimed to evaluate colonic inflammation through mRNA levels of different pro-inflammatory cytokines. Compared to the CTRL group, the ABA-C group exhibited no alteration of IL-1β, TNF-α, and TLR4 mRNA, whereas the MCP-1 mRNA level was markedly decreased (Supplementary Figure S4). Interestingly, refeeding partially restored the MCP-1 mRNA level without adding effects of amino acids. Refeeding did not affect IL-1β, TNF-α, and TLR4 mRNA level. Only the ABA-Rf-G group exhibited an increase in IL-1β mRNA, while BCAA had no influence (Supplementary Figure S4).

## 4. Discussion

In the present study of mice subjected to the activity-based anorexia, we report that: (i) Progressive refeeding is associated with a partial restoration of body weight with a maintained altered body composition, (ii) both Gln and BCAA-supplemented refeeding fail to improve body composition and body weight, and (iii) Gln-supplemented refeeding, but not BCAA-supplemented, is associated with a mTOR-mediated increase in colonic protein synthesis. Optimizing the refeeding remains a major issue in AN patients to limit malnutrition-induced complications and avoid frequent relapses. Indeed, mood and gastrointestinal functional disorders frequently observed in AN patients are not completely resolved after body weight gain [36], which can also participate in the chronicization of AN. In the last decades, the role of microbiota-gut-brain axis has been highlighted in the regulation of mood and gastrointestinal disorders, as well as in the regulation of food intake [10,37]. In addition, more recently, the microbiota-gut-muscle axis appears of interest to limit the loss of muscle mass observed in chronic diseases such as cancer [38]. Therefore, targeting the gut-brain and gut-muscle axis could be of interest in AN patients.

AN patients exhibited a marked decrease in both fat and fat-free mass and body weight gain is associated with an altered fat tissue distribution, particularly with an increase in abdominal fat mass contributing to maintain body shape perception alteration [39]. In the present study, and as previously described [25,30], both fat and fat-free mass are reduced in the ABA model. We previously reported that a short-term refeeding induced a fat mass rebound, while fat-free mass remains lower than in controls [25]. In this latter study, refed mice had free access to food leading to hyperphagia, which is not observed in clinical practice. Therefore, we decided to develop a progressive refeeding protocol, as described in the Methods Section. The access to the running wheel was maintained during the refeeding period, as beneficial effects of adapted physical activity have been underlined [17]. Interestingly, after 10 days of progressive refeeding, the body weight and lean mass were not completely restored, while the fat mass rebound remained present. The cumulative food intake during the refeeding period was higher in refed mice than in non-refed mice, but similar to the control mice. The hypothalamic NPY/POMC ratio, which is an adaptive mechanism to undernutrition, was partially restored according to the improvement of body weight and the increase of food intake. Anorectic patients are known to suffer from intestinal disorders such as delayed gastric emptying, which made them feel bloated [40] and consequently, could impair refeeding protocols. Here, we observed a decreased gastric emptying in ABA-C mice compared to CTRL, as previously described, [18] which was restored by refeeding. However, the drop was less marked compared to our previous work [18]. We hypothesized that this difference could be explained by a sex difference, as in the present study, and we previously used male instead of female mice [18]. In a separation-based model of anorexia, refeeding was associated with an increase in fat mass without restoration of plasma leptin [41]. However, during a refeeding program in AN patients, plasma leptin increased [42,43] and was positively associated with the body mass index changes [44]. Interestingly, in the ABA model, progressive refeeding was associated with the restoration of glycaemia, as well as plasma leptin. Therefore, in our opinion, this refeeding protocol well mimics the nutritional care of AN patients and can be used to evaluate the capacity of specific nutrients, such as amino acids, for improving body weight gain, body composition, and intestinal functions.

Among amino acids, we focused on Gln and BCAA due to their respective impact on intestinal functions and body composition. Indeed, Gln is well known to regulate protein intestinal metabolism [27], inflammatory response [28], and intestinal permeability [19]. By contrast, BCAA has been proposed to promote muscle protein synthesis and lean mass. M Holecek recently reviewed the impact of BCAA on protein metabolism and showed that BCAA is able to stimulate the muscle protein synthesis through the mTOR pathway and limit muscle protein degradation [45]. In a previous work, we showed that both Gln and BCAA supplementations failed to improve body weight [30], which could be explained by a lack of energy supply in anorectic mice. In the present study, we combined progressive refeeding to Gln or BCAA supplementation. Gln supplementation was able to improve neither body weight gain nor body composition. BCAA supplementation tended to improve body weight gain (*p* = 0.06) mainly by increasing fat mass (*p* = 0.06). By contrast, BCAA supplementation induced neither a gain of lean mass nor muscle protein synthesis, which was expected. In older rats, opposite results have been published: 4.5% of leucine during 6 months induced adipose tissue hypertrophy and hyperplasia [46], while 4% of leucine for 40 weeks limited fat mass gain [47]. In those studies, leucine supplementation did not affect muscle mass. We did not measure muscle protein degradation, which should be further explored in ABA mice. In addition, in patients with liver cirrhosis, BCAA supplementation was associated with a decrease of intramuscular fat mass only in patients with restored plasma albumin [48]. Hypoalbuminemia only occurs in severe AN patients and ABA mice did not exhibit an alteration of plasma albumin. Finally, both Gln and BCAA supplementations did not affect the hypothalamic NPY/POMC ratio suggesting that those amino acids did not regulate the feeding behavior in our tested conditions. All these data showed that both Gln and BCAA supplementations failed to appropriately improve body composition in the ABA refed mice.

In previous studies, we reported alterations of colonic response in ABA mice with increased paracellular permeability [25,31] and altered protein metabolism [35]. Indeed, ABA mice showed a reduction of protein synthesis and an activation of autophagy related to an inactivated mTOR pathway [35]. This response is specific for colonic tissue compared to hypothalamus [34]. In the present study, ABA-C mice did not exhibit an increased colonic paracellular permeability conversely to our previous studies [25,31]. This discrepancy could be explained by the different time course: Day 23 in the present study compared to day 17 in previous studies. A progressive refeeding was associated with an improvement of protein synthesis and a restoration of autophagy. Interestingly, Gln supplementation enhanced protein synthesis restoration through an activation of mTOR pathway, particularly phosphorylation of p70S6kinase. Surprisingly, BCAA supplementation blunted colonic protein synthesis improvement and limited the inhibition of autophagy. An increase in protein synthesis in response to leucine has been shown in intestinal epithelial cells [49]. However, in healthy volunteers, the leucine supply was not able to stimulate duodenal protein synthesis [50]. Nevertheless, in addition, while Gln tended to improve tight junction protein expression, CLDN-1 and OCDN, the BCAA supplementation blocked the effects of refeeding on those parameters. These data are in accordance with our previous study showing that Gln was able to restore colonic paracellular permeability and protein synthesis during the underfeeding period in ABA mice, while BCAA had no effects [30]. Further studies should be done to decipher the underlying mechanisms.

Our study has some limitations. We used male mice while AN is a female predominant disease. However, male and female mice have different responses to the ABA model [51]. Particularly, females exhibit an earlier adaptive response to the ABA model than males. The ABA procedure needs to be adapted to female mice before evaluating the effects of refeeding. Finally, in the present study, we did not assess the psychopathological aspects of AN, which can be affected by the kinetics of weight changes [52] and need to be further investigated.

In conclusion, glutamine and branched-chain amino acids supplementation during refeeding fail to expectedly improve body composition in ABA mice. Glutamine, but not branched-chain amino acids, shows beneficial effects on colonic protein metabolism. Further studies should evaluate whether glutamine effects are associated or not with an improvement of gastrointestinal functional disorders.

## Supplementary Materials

The following are available online at https://www.mdpi.com/2072-6643/12/11/3510/s1, Figure S1: Body weight, Figure S2: Muscular protein synthesis and tight junctions mRNA expression, Figure S3: Gastric emptying, Figure S4: Colonic inflammatory markers, Table S1: List of primary and secondary antibodies used in western-blotting, Table S2: List of primers used in RT-qPCR.
