# Supplementary material for: Influence of Glutamine and Branched-Chain Amino Acids Supplementation during Refeeding in Activity-Based Anorectic Mice
Source: Nutrients. 2020 Nov 14;12(11):3510. doi: 10.3390/nu12113510 (PMC7696484; doi:10.3390/nu12113510)
Supplement: Supplementary file 1 [file nutrients-12-03510-s001.pdf]

SUPPLEMENTAL FIGURES

Table S1. List of primaries and secondaries antibodies used in western-blotting.

Proteins	References	Species	Dilution
**Actin**	Sigma-Aldrich; A5441; Saint Louis; MO; United States	Mouse	1 : 5000
**Claudin-1**	Invitrogen; MH25; Carlsbad; CA; United States	Rabbit	1 : 1000
**GAPDH**	Sigma-Aldrich; SAB2500541; Saint Louis; MO; United States	Goat	1 : 5000
**LC3B**	Novus Biologicals; NB100-2200; Littleton; CO; United States	Rabbit	1 : 1000
**Occludin**	ThermoFisher Scientific; OC-3F10; Rockford; IL; United States	Mouse	1 : 500
**p70S6K**	Cell Signaling; 9202S; Danvers; MA; United States	Rabbit	1 : 500
**p-P70S6K**	Cell Signaling; 9205S; Danvers; MA; United States	Rabbit	1 : 1000
**Puromycin**	Merck Millipore; MABE343; clone 12D10; Darmstadt; Germany	Mouse	1 : 5000
**TLR-4**	Santa Cruz; SC-30002; Dallas; Tex; United States	Rabbit	1 : 1000
**Anti-Goat**	Dako; P0160; Santa Clara; CA; United States	Rabbit	1 : 5000
**Anti-Mouse**	Dako; P0161; Santa Clara; CA; United States	Rabbit	1 : 5000
**Anti-Rabbit**	Dako; P0399; Santa Clara; CA; United States	Swine	1 : 5000

Table S2. List of primers used in RT-qPCR.

Genes	Sens	Sequences
**Claudin-1**	F	5’-CTGGGTTTCATCCTGGCTTC-3’
	R	5’-TTGATGGGGGTCAAGGGGTC-3’
**Claudin-2**	F	5’-ATACTACCCTTTAGCCCTGACCGAGA-3’
	R	5’-CAGTAGGAGCACACATAACAGCTACCAC-3’
**CRH**	F	5’-AGAAGAGAGCGCCCCTAAC-3’
	R	5’-ATCAGAATCGGCTGAGGTTG-3’
**GAPDH**	F	5’-CATCACTGCCACTCAGAAGA-3’
	R	5’-AAGTCACAGGAGACAACCT-3’
**Il-1β**	F	5’-CCCAAAAGATGAAGGGCTGC-3’
	R	5’-AAGGTCCACGGGAAAGACAC-3’
**MCP-1**	F	5’-TTAAAAACCTGGATCGGAACCAA-3’
	R	5’-GCATTAGCTTCAGATTTACGGGT-3’
**Muc-2**	F	5’-CGACACCAGGGATTTCGCTTAAT-3’
	R	5’-CACTTCCACCCTCCCGGCAAAC-3’
**NPY**	F	5’-CTGCGACACTACATCAATCT-3’
	R	5’-CTTCAAGCCTTGTTCTGG-3’
**Occludin**	F	5’-AGACTACACGACAGGTGGGG-3’
	R	5’-CTGCAGACCTGCATCAAAAT-3’
**POMC**	F	5’-CCTCCTGCTTCAGACCTCCA-3’
	R	5’-GGCTGTTCATCTCCGTTGC-3’
**TNF-α**	F	5’-TGTCTACTCCTCAGAGCCCC-3’
	R	5’-TGAGTCCTTGATGGTGGTGC-3’
**ZO-1**	F	5’-GCAGACTTCTGGAGGTTTCG-3’
	R	5’-CTTGCCAACTTTTCTCTGGC-3’
**β2m**	F	5’-GCCGAACATACTGAACTGCTAC-3’
	R	5’-GCTGAAGAACATATCTGACATCTC-3’
**RPS18**	F	5’-TGCGAGTACTCAACACCAACA-3’
	R	5’-TTCCTCAACACCACATGAGC-3’

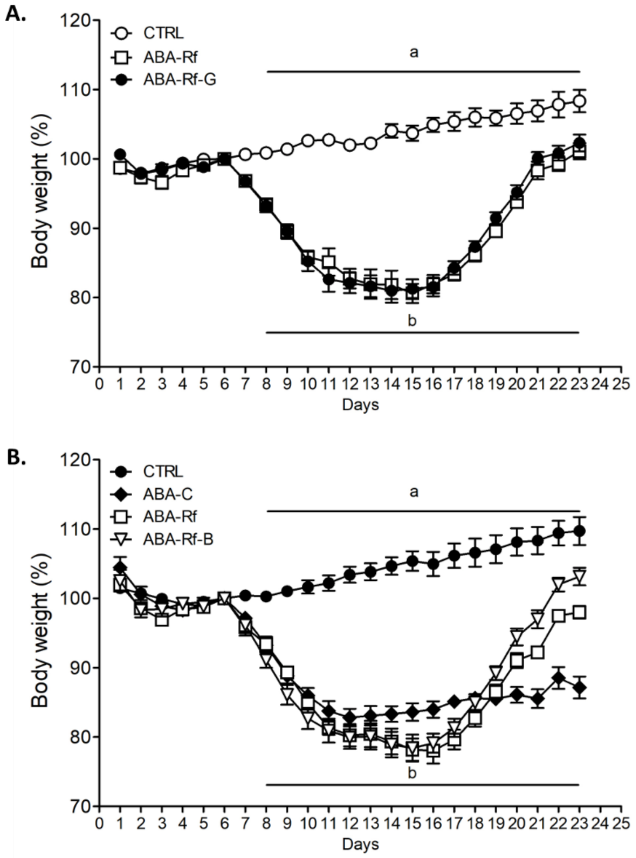

Figure S1: Body weight. Body weight was monitored each day in both experiments, glutamine –supplemented (A) and branched chain amino acids-supplemented (B), in control mice (CTRL), in activity-based anorexia (ABA) mice (ABA-C) and in ABA mice after refeeding supplemented or not (ABA-Rf group) with 1% glutamine (Gln, ABA-Rf-G group) or 2.5% branched chain amino acids (BCAA, ABA-Rf-B group). Data were compared with two- way ANOVA followed by Bonferroni posttests. Values without a common letter significantly differ ($p < 0.05$). n=5-8/per group.

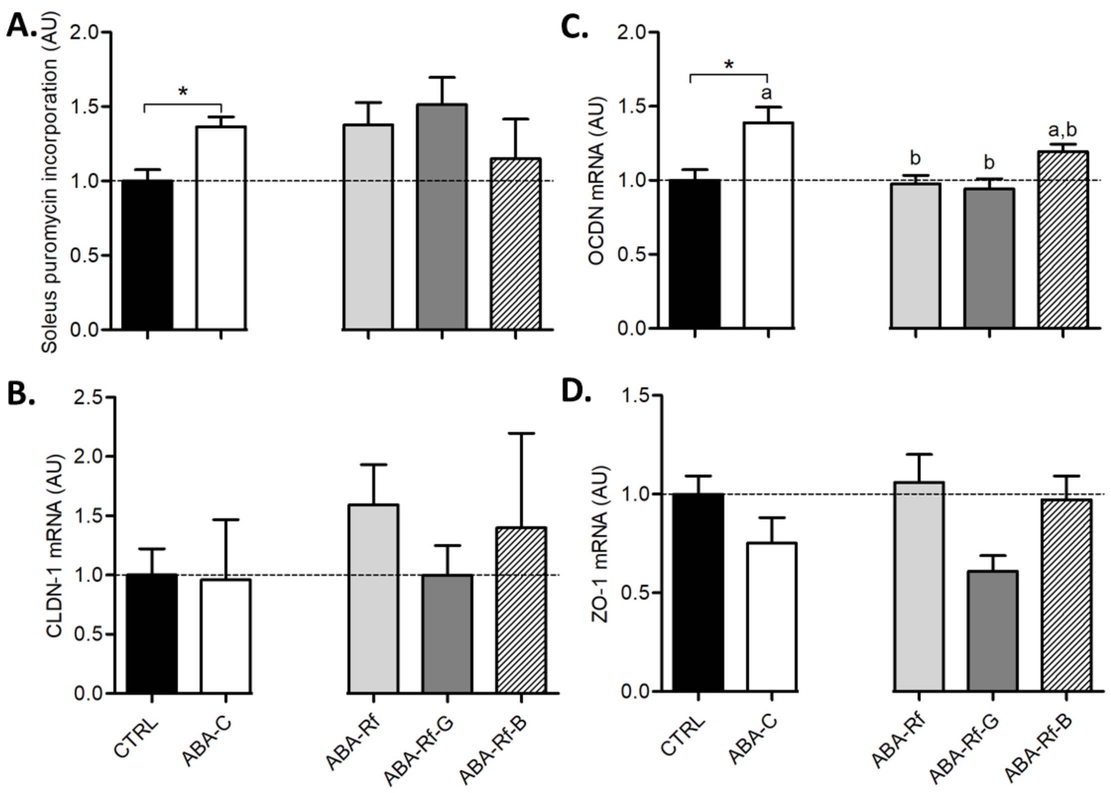

Figure S2: Muscular protein synthesis and tight junctions mRNA expression. Soleus Protein synthesis (**A**), and colonic claudin-1 (CLDN-1, **B**) occludin (OCDN, **C**) and zonula occludens-1 (ZO-1, **D**) mRNA levels measured at day 23 in control mice (CTRL), in activity-based anorexia (ABA) mice (ABA-C) and in ABA mice after refeeding supplemented or not (ABA-Rf group) with 1% glutamine (Gln, ABA-Rf-G group) or 2.5% branched chain amino acids (BCAA, ABA-Rf-B group). Data from CTRL and ABA-C groups were compared by Mann-Whitney test, *, $p<0.05$. Then, ABA-C and refed Groups were compared with ANOVA followed by Tukey posttests. Values without a common letter significantly differ ($p < 0.05$). n=5-8/per group.

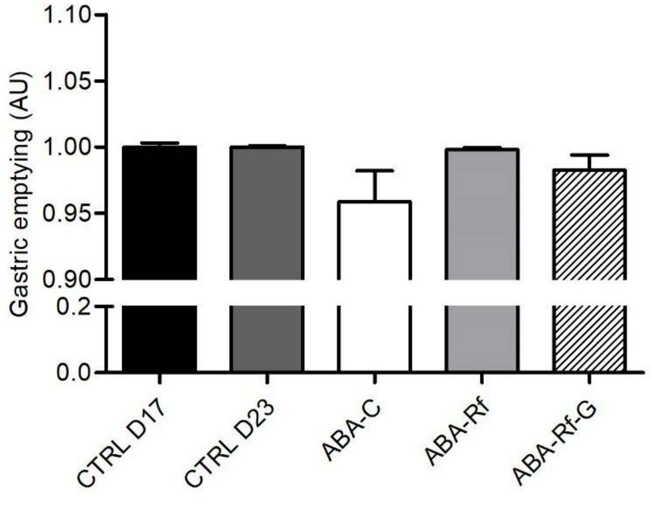

Figure S3: Gastric emptying. Gastric emptying measured at day 17 and day 23 in control mice (CTRL), in activity-based anorexia (ABA) mice (ABA-C) and in ABA mice after refeeding supplemented or not (ABA-Rf group) with 1% glutamine (Gln, ABA-Rf-G group). n=5-8/per group.

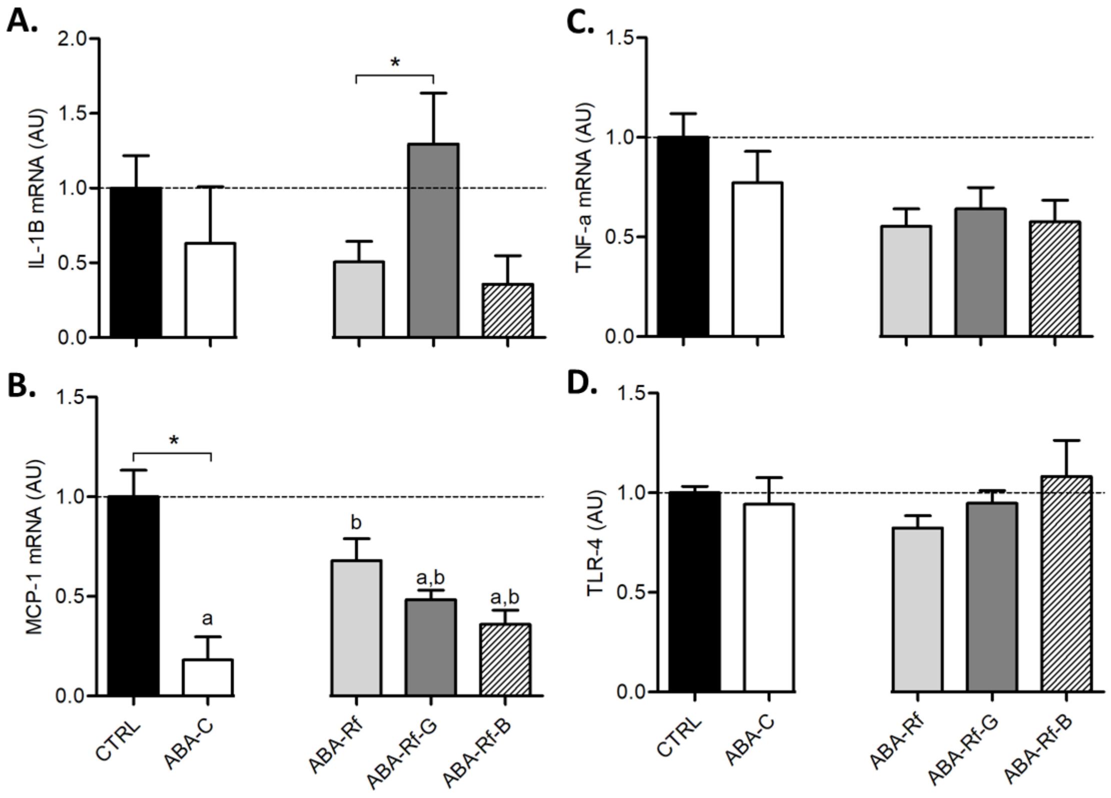

Figure S4: Colonic inflammatory markers. IL-1β (A.), MCP-1 (B.), TNF-α (C.) and TLR4 (D.) mRNA levels measured at day 23 in control mice (CTRL), in activity-based anorexia (ABA) mice (ABA-C) and in ABA mice after refeeding supplemented or not (ABA-Rf group) with 1% glutamine (Gln, ABA-Rf-G group) or 2.5% branched chain amino acids (BCAA, ABA-Rf-B group). Data from CTRL and ABA-C groups were compared by Mann-Whitney test, *, $p<0.05$. Then, ABA-C and refed Groups were compared with ANOVA followed by Tukey posttests. Values without a common letter significantly differ ($p < 0.05$). n=5-8/per group.
